# The Referee as an Educator: Assessment of the Quality of Referee–Players Interactions in Competitive Youth Handball

**DOI:** 10.3390/ijerph17113988

**Published:** 2020-06-04

**Authors:** Katarzyna Płoszaj, Wiesław Firek, Marcin Czechowski

**Affiliations:** Faculty of Physical Education, Józef Piłsudski University of Physical Education in Warsaw, 00-968 Warsaw, Poland; wieslaw.firek@awf.edu.pl (W.F.); marcin.czechowski@awf.edu.pl (M.C.)

**Keywords:** referee, referee–players interactions, handball, educational practice, pedagogical function, youth sport

## Abstract

Sport does not automatically generate educational benefits for players. For a sports field to become a child-friendly educational environment, it is essential that all actors involved in the organization of youth sport take deliberate educational measures. Among these actors are referees, who should be taken into account during the research on the educational value of sport for the youngest. The subject of the present study was handball referees, who interact with the players during matches. Assuming that the referee is an important actor in sport education and that referee–players interactions are the basic mechanism of the referee’s educational influence, this study aimed to assess the quality of his or her interactions with players during handball matches for children aged 9 to 12 years. The research was conducted in a group of 25 handball referees who refereed matches of children in the region of Mazowieckie Voivodeship in Poland. The referees surveyed had current licenses issued by the Warsaw–Mazovian Handball Association. To assess the quality of referee–players interactions, the authors’ direct observation tool (Referee–Players’ Interaction Assessment Scoring System) was used. The educational referee–players interaction was studied in six dimensions: Positive climate, Responsiveness, Behavior management, Proficiency, Instructing, and Communicating. Data were statistically analyzed using chi-squared test, Mann–Whitney U test and exploratory factor analysis (EFA). Cronbach’s alpha values were higher than 0.90 in the factors, showing adequate levels of reliability. The results of the research demonstrated that the assessment of the quality of the referee’s educational influence on players was neither affected by the referees’ experience nor by the outcome of the match. The quality of educational referee–players interactions in five of the six dimensions studied was assessed as average, whereas positive climate was assessed as poor (three-step scale: poor, average, good). If referees are to support coaches and parents in achieving their educational goals, the results indicate areas where they can improve. The research provided empirical evidence that could be used as a basis for the modification of previous training programs for referees developed by local and national sports associations. The referees should be trained to build a positive climate on the sport field, which consists in creating emotional ties with players (physical proximity, social conversation), expressed in an enthusiastic attitude and joy of contacts (smiling, engagement, positive affect reaction, positive comments, respectful and inclusive language, using players first names, listening to players). In addition, referees must be taught to actively monitor players’ emotional, cognitive, social, and health needs, as well as to respond to the players’ needs and solve problems.

## 1. Introduction

Many actors and institutions are involved in the organization of youth sport. Some of them undertake long-term deliberate initiatives aimed at the cognitive, moral and social development of players, using people with appropriate education (pedagogical and psychological). Others treat the educational process as an additional activity, undertaken more or less consciously. For a sports field to become a child-friendly educational environment, it is essential that all actors who organize youth sport take deliberate educational measures.

Various educational strategies for sport [1,2,3,4], normative models of the coach [5,6,7,8], parents [9,10,11] and teachers of physical education [12,13,14], and tools for the assessment of social and antisocial behavior of players [15,16,17] have been developed so far. Shields et al. [18] emphasized that sport does not automatically generate educational benefits for children and young people. It is the behavior and attitudes of adults during training sessions, matches, training camps, etc. that are critical to their multilateral development. Athletes often imitate the behavior of their coaches, parents, or teachers, and take their attitudes, views, norms, and values as their own [19]. Therefore, all adults involved in youth sport are teachers. These adults also include referees, who are often forgotten in studies of the educational values of youth sport.

Publications on the sports environment as a social space [20] show that sport has educational potential [21]. If properly and skillfully used by coaches, parents, or referees, it can lead to the multifaceted development of the personality of young players. It is often stressed that participation in sport improves children’s self-esteem [22], helps maintain emotional balance, and encourages cooperation and leadership [23,24]. Training, sports competition, losing, and winning can be a rich source of positive personal and social experiences provided that these situations are skillfully and consciously used for educational purposes [25,26,27,28,29,30,31]. In addition, research results relating to the referees’ use of techniques such as modelling, adopting child perspective, redirecting inappropriate behavior, building a positive climate, etc. increase the effectiveness of educational effects [32,33,34].

According to Andersson [35], ‘referees and refereeing is an under-explored field of research (…) and the pedagogical function of referees is one of key importance in the co-creation of educational practices’ (pp. 616–617). In scientific literature, the educational function of the referee is usually enumerated after the supervisory and ordering function. While the educational function is limited in professional sport, it should be of key importance, or at least equivalent to other functions, in youth sport. Adopting such a perspective makes the referee perceived not only as a sports competition controller but also as a teacher with adequate knowledge of pedagogy and developmental psychology [36]. A referee who is the only adult on the pitch during competitions in youth sport can make his or her attitudes and decisions an important source of stimuli shaping prosocial behavior, value systems, knowledge, and skills of young players. He or she also influences the attitudes of spectators, coaches, and other sports officials [35]. Therefore, referees should be viewed not only as ordinary technicians and evaluators of performances in competition [21], but also as teachers.

The problems of the educational function of a referee are increasingly becoming the subject of academic reflection. Among other things, this is because sports governing bodies, supporters, and parents of young players require increasingly higher qualifications and competences from referees. To date, extensive research has been undertaken on various aspects of the work of referees, including: (a) the determinants of their decisions [37,38,39,40,41]; (b) expected physical fitness in referees [42]; (c) identification of the key competencies of the referee [43,44,45]; (d) officiating communication [46,47,48,49,50,51]; and (e) athletes’ expectations with regard to officiating competence [46,52,53]. Nevertheless, MacMahon et al. [43] argue that there are relatively few such studies. These authors indicated the diversity of roles that referees play in different sports as a major barrier to such studies. Different requirements have to be met by a handball referee and something else is required from a judge in gymnastics or a line umpire in tennis. In addition to controlling the actual play during a competition, sports officials must have the positive characteristics of a police officer, lawyer, judge, accountant, reporter, athlete, and diplomat.

Simply appealing to the bodies responsible for training referees to emphasize the educational dimension of refereeing is insufficient. Such demands should be thoroughly supported by empirical scientific evidence. The referees need more than just textbooks with rules of the game because nowadays they are required to show communication skills or build positive relationships with players [47]. However, as Dosseville, Laborde, and Bernier [52] argue, the interpersonal dimension of the athlete–official relationship remains largely unexplored. Lack of referees’ knowledge and skills to build a positive climate during a match or to prevent negative player’s behavior can contribute to children leaving the sport [1,34,54,55].

In addition, justification for undertaking this research problem is lacking a comprehensive analysis (and diagnosis) of the mutual relations between the adult actors involved in youth sport and players during sports competitions. Kirk, Macdonald, and O’Sullivan [56] stressed that sport pedagogy has three key and interlinked elements: learning, teaching, and curriculum. Over the past 20 years, research has focused mainly on the contents (curricula) and the activities of educators. Consequently, empirical research in the field of sport pedagogy did not often focus on the interactions between the educator (coach, parent, referee) and the learner (player). The presented research fits into the basic objectives of sport pedagogy, namely, to show the possibility of using sport as a means of education and to search for ways to increase the effectiveness of educational effects of sport [57].

## 2. Purpose

Taking into account that referees play different roles in different sports, MacMahon and Plessner [58] enumerated three types of interactions between referees and players: (i) those who interact (handball, soccer, basketball, boxing), (ii) those who react (tennis, volleyball, track and field), and (iii) those who judge (artistic gymnastics, artistic swimming, figure skating). The focus of the present study is on handball referees, who interact with players in many ways. Based on the assumption that the referee is an important actor in sport education, and that referee–players interactions are the basic mechanism of his or her educational influence, this study aimed to assess the quality of the referee’s interactions with players during handball matches for children aged 9 to 12 years. The research goal formulated in this way first requires developing a referee–educator normative model and constructing a tool enabling the assessment of referee–player educational interactions. To check whether other factors influence the assessment of the quality of the referee–player interaction, the following null hypothesis was proposed: The experience of the referees and the results of the matches which they refereed do not affect the assessment of the quality of their educational interactions with the players. For the research, it was assumed that an experienced referee is one who had refereed matches for two years and more. In this study, it was assumed, that a clear advantage of one of the teams means the goal difference was ≥3. In a close match the goal difference was <3.

This assessment can help identify the strengths and weaknesses of the referee’s work. The results of the research can be used to raise the referees’ awareness of the importance of their relations with players on the pitch for the educational practice in sport. The conclusions from the research can also be useful for developing or modifying training programs for sports referees.

## 3. Materials and Methods

### 3.1. Participants

The research was conducted in a group of 25 handball referees who refereed matches of children aged 9 to 12 years in the region of Mazowieckie Voivodeship in Poland. The referees surveyed had current licenses issued by the Warsaw–Mazovian Handball Association. The research involved 19 men and six women. The mean age of the participants was 24.64 years (standard deviation (SD) = 9.98 years), with an age range of 17–51 years. The mean number of years of experience as a referee was 5.28 years (SD = 7.24 years, ranging from 1 to 23 years). The referees were observed as they officiated, during the period of the study, handball matches of children organized by district sports associations.

### 3.2. Measures

The authors’ direct observation tool, Referee–Players’ Interaction Assessment Scoring System (R-PIASS), was used to assess the quality of referee–players interactions. This tool has been developed based on the literature review and empirical research on the role of a referee in sport and teacher–student interactions. The basis for the development of the R-PIASS tool was the assumption that the educational function of the referee is expressed in the quality of interactions with young players. The referee–players interactions were divided into six dimensions. The description of the individual dimensions is presented in Table 1.

### 3.3. Developing R-PIASS Dimensions

#### 3.3.1. Positive Climate

The basis for a child’s positive experience in sport is a good relationship with peers, the coach, and the supporting parents. The person who is not without significance for the climate during the game is the referee. For sporting competitions to become safe and educational events for children, all those involved in the organization of children’s sports must work together and create a positive climate. Nowadays, it is stressed that youth sport should be free from competition and striving for winning at all costs [14]. A sports match should be a source of joy and satisfaction, because only in such an atmosphere can a child develop properly [7]. The International Charter of Physical Education, Physical Activity and Sport (UNESCO) stresses that physical education, physical activity, and sport should aim to promote stronger interpersonal ties, solidarity, mutual respect, and understanding, and to preserve the dignity of every human being. Studies suggest that with the rejection of these values, the withdrawal of children from physical activity increases [59]. Engh [60] claims that about 70% of children leave sports after the age of 13 years. These decisions result primarily from the lack of time to play, excessive pressure on competition and victory, and negative experiences in interpersonal relations [14,33,34,36,54,59]. Another reason for children’s withdrawal from sports is the excessive pressure exerted by the coach [55] or parents [11].

There is a dominant conviction in the field of pedagogy that proper teacher–student interactions are the key to positive learning outcomes and instilling the prosocial behavior of the students. Pupils achieve improved results in demanding but supportive environments [61,62]. Noddings [63,64] emphasized that the school should primarily create a caring climate. If students view it as a friendly educational environment, they develop better in the physical and emotional sphere [65,66]. The same approach is currently being transferred to the area of sport [67] and has been reflected, among others, in the concept of positive youth development through sport, which postulates that youth sport should be oriented towards stimulating positive experiences based on enjoying participation in sport [1,32,33,68].

If one considers that a positive climate is one of the main determinants of sporting experience of young players, all actors involved in youth sport should take deliberate actions aimed at building a positive atmosphere of the game. The mental status of players is particularly influenced by the behavior of the referee, who can generate tension and interpersonal conflicts during the match [69]. The strategies for building a positive climate are different. Fry [67] offers the following techniques to coaches: setting the tone from day 1; provide a warm and friendly greeting to each participant; provide a positive welcome to the entire team; setting clear expectations for all to hear; helping athletes build relationships with one another; and bringing parents on board. The referee should also skillfully apply techniques of building a positive climate, e.g., give young athletes positive comments on their achievements and correct behavior [8,70]. Other characteristics of the desirable behavior of the referee are enthusiasm and smile, physical closeness, and respect for the players [71]. If the referee does not feel responsible for building a positive climate, then according to the *primum non nocere* principle, he or she should not at least build a negative one.

#### 3.3.2. Responsiveness

The emotional support of a young athlete also requires the ability to observe and recognize his or her emotional states [37]. In the opinions of referees, the ability to interpret the players’ emotions and behaviors characterizes every good referee. This aspect of the referee’s work has been termed situation monitoring. It can also be called reading and interpreting player [47]. The dynamic situation on the sports field, the atmosphere of competition, and the pressure of the result make it necessary for the referee to be constantly alert. In addition to looking for opportunities to support players’ emotional, cognitive, behavioral, social, and health needs [10,36], the referee should respond adequately to the situation to solve problems as quickly as possible and prevent them from escalating over time. To act effectively in this regard, the referee must take the child’s perspective and help children to take the others’ perspectives [36,72]. Since each match is different, referees cannot always use the same educational means, methods, and techniques. Different situations on the pitch require the use of an appropriate refereeing style and matching it with the atmosphere of the match. This is particularly important in invasion games where the referee’s decisions cannot be a strict application of the letter of the law [73,74,75]. Refereeing requires creative and fair solutions beyond the rulebook [43].

#### 3.3.3. Behavior Management

An important area where the referee’s work can be described and evaluated (from the educational perspective) is game organization. In the literature, this area is most often described alongside the psychological aspects of decision making. Game organization is related to the actions taken by the referee before the sporting event (checking the facilities, greeting the athletes and coaches), during the event (refereeing), and after the event (writing match reports) [43]. Since the present study deals with the educational aspect of the referee’s work in youth sport, the focus is on how referees prevent negative behavior and how they redirect this behavior to a socially acceptable one. In general, this means how they manage the players’ behavior during the game. As Macmahon [43] notes, refereeing is more like practicing problem-solving and less like decision-making. From a pedagogical point of view, a sporting event will be conducive to education when the level of aggression and the number of unsportsmanlike behavior instances can be minimized. The referee should manage the behavior already before the match during the first contact with players or team captains. This is a good time to present the expectations concerning the players’ behavior during the game and to set clear boundaries that should not be transgressed [10,43,76]. The referee’s consistency in the case of behavior not meeting previous arrangements is also essential. Negative behavior is less likely when punishment is imminent [36]. The referee should use preventing officiating and preventing communication [38], and when these actions are ineffective, he or she should redirect negative behavior using appropriate methods. The study [32,33,34,36] demonstrated that referees knowing and using techniques of shaping pro-social behavior were much more likely to be able to effectively manage the game and strengthen the positive behavior of players compared to those who failed to use such techniques. Even brief modeling is more effective than no modeling.

#### 3.3.4. Proficiency

In this area, the ability of the referee to organize the game smoothly and without unnecessary interruptions is important. All players should know what they are expected to do. An indicator of professionalism and proficiency in this aspect is also the proper preparation of the referee for the sporting event in terms of his or her knowledge and the necessary refereeing equipment [43].

#### 3.3.5. Instructing

There are descriptions in the literature concerning the different dimensions of a referee’s work and their refereeing styles. In addition to the ‘dictator’ referee and the laissez-faire style, Macmahon [43] enumerates the ‘teacher’ referee, who clearly and precisely justifies his or her decisions to prevent repeated violations of the rules of the game. The benefits of the development of the prosocial behavior of young athletes resulting from instruction were described by Berkowitz and Grych [10]. Instructing players consists in providing information about sports rules (helping to interpret them) and teaching the value of sport. Activities related to the clarification of existing rules of the game are particularly important in youth sport.

#### 3.3.6. Communicating

It is worth noting that “effective communication at times can be more important than the decisions themselves” [43] (p. 81). Simmons [77] claims that even wrong decisions can be positively perceived by the players if they are communicated appropriately. Furthermore, the athletes themselves consider referees to be more honest if they explain their decisions and express them with a calm voice [49]. The referee’s messages should be concise, short, and spoken slowly [47]. During a match, there is often no time to talk to the players. Referees mostly use one-way communication and therefore they are primarily responsible for the quality of communication [78]. First, the referee expresses his/her decisions with a whistle and a hand signal and then verbally. Effective communication starts with the effective method of blowing the whistle and the quality of gestures. The knowledge of using the whistle and proper signaling mechanics is fundamental for communication and education of young athletes [47]. A description of the individual dimensions and indicators of the R-PIASS tool and the way of determining their scores is shown in Table 2.

## 4. Procedures

The research was approved by the Research Bioethics Commission of the Józef Piłsudski University of Physical Education in Warsaw (SKE 01–10–2020). Before starting the research, the research team met with the authorities of the Warsaw–Mazovian Handball Association (Warsaw, Poland) to present the objectives and tools used in the research and received permission to conduct it. All the referees were informed by the sporting association about the planned examinations. Immediately before the observation, all survey participants received information about the objectives of the survey, voluntary participation, and anonymity of results. Each referee had to sign a consent form to participate in the study.

The research consisted of a one-time direct observation of the referee’s work during a match for children aged 9–12 years by trained observers, who were the authors of the R-PIASS tool. The relevant studies were preceded by the pilot research to test the tool and to standardize the criteria for assessing the quality of the referee’s interaction with the players. The observers took part in three test observations. It was assumed that the conformity of the referee’s work assessment should be above 80%. This allowed for minimization of the subjectivity of the assessments and enabled statistical analysis of research results. Each observation covered the period immediately before, during, and immediately after the match. The observation took place in a controlled manner, i.e., it consisted in regular live observation of the referee–players interactions according to a strictly defined R-PIASS key. The actions of each referee were evaluated by one observer, who noted them on the R-PIASS score sheet. Each time immediately after the observation was completed, the researcher assessed the referee’s work based on score sheet notes by comparing them to the description of individual dimensions of the R-PIASS tool (Table 2). Each dimension was evaluated on a 7-step scale. How the assessment of a given dimension is determined based on indicators is illustrated in Table 3.

## 5. Analytic Strategy

Basic statistical measures were used to describe the results: arithmetic means, standard deviations, and medians. The Mann–Whitney U tests were used to determine the differences in the group of referees. Cronbach’s alpha coefficient was calculated to assess the consistency of the analyzed variables. Exploratory factor analysis (EFA), with principal axis factoring (PAF), was used to identify the underlying relationships between variables. The Kaiser–Meyer–Olkin measure of sampling adequacy (KMO) and the Bartlett’s test of sphericity were run to check that the data were appropriate for EFA. An orthogonal Varimax rotation was used for factor rotation. For item reduction, the cut-off for significance of factor loading was set to 0.5. Calculations were performed using the PASW Statistic 18 (IBM Corp., Armonk, NY, USA).

## 6. Results

In the survey, 68% of the referees were characterized by refereeing experience of less than two years. About 72% of the analyzed matches took place with a clear advantage of one of the teams (a goal difference ≥3) while such matches were equally often officiated by referees of more or less experienced (χ^2^(1) = 0.991; *p* > 0,05). Analysis of the results of experienced referees in comparison with inexperienced referees showed no significant statistical differences between the studied groups in the quality of interaction in all six dimensions (Table 4). The test (Table 5) revealed that there were no statistical differences in the assessment of referee–players interactions during a close match or a match with the advantage of one of the teams. Therefore, the results presented in Table 6 do not take into account the division into groups.

Means and standard deviations for all examined dimensions are reported in Table 6. Positive Climate was rated as poor ($\bar{x}$ = 2.8; SD = 1.00). The quality of the referee–players interaction in this dimension was rated worse than the others. It is worth noting that almost half of the referees (48%) were rated as average ($\bar{x}$ = 3.0), and one referee was rated as good. The Responsiveness dimension was assessed on an average level. The quality of interactions of six referees with players (24%) was assessed as poor in this dimension and two (8%) were rated good. Two more dimensions: Behavior management ($\bar{x}$ = 4.7; SD = 1.5) and Proficiency ($\bar{x}$ = 5.0; SD = 1.5) were rated as average. The second was rated the highest of all dimensions. Nearly half of the referees surveyed received good scores in this respect. The quality of referee–players interactions was also assessed at a medium level in the dimensions of Instructing ($\bar{x}$ = 4.5; SD = 1.6) and Communicating ($\bar{x}$ = 3.9; SD = 1.4).

The value of Cronbach’s alpha of 0.92 obtained from the analyses indicates a high similarity of the assessments taken into account in the study. The lowest values of correlation with the overall result were found for the Positive climate (0.463). Other ratings correlated much more strongly with the total scale, with the coefficients ranging from 0.776 (Responsiveness) to 0.894 (Behavior management).

Factor analysis was made for six variables. The KMO measure was at an acceptable level (0.786) and Bartlett’s sphericity test was also significant (*p* < 0.001), indicating the validity of the factor analysis. In the initial phase of factor analysis, one eigenvalue was ≥1, thus meeting the Kaiser criterion when determining the number of factors. However, no clear and easy in interpretation structure was obtained for the one-factor solution, explaining only 71.753% of the variance (Table 7). Therefore, with a small number of variables included in the analysis, the number of factors in the second approach was determined using Cattell’s criterion (scree plot) and the percentage of explained variance (>0.8). In this solution, two factors were identified, as described below.

Table 8 shows the factor loadings after Varimax rotation using a significant factor criterion of 0.5. The choice of cut-off depended on the ease of interpretation. The results of the exploratory factor analysis (EFA), including two factors (Table 8), showed that first factor created variables: Behavior Management, Proficiency, and Instructing with quite high factor loadings (>0.903), Responsiveness (0.637) and Communicating (0.614), however, two assessments contributed equally to the loadings of Factors 1 and 2. Responsiveness and Communicating were complex variables. The second factor contained only a variable Positive climate. This indicates, similarly to the results of the analysis of Cronbach’s alpha, a slightly different character of this variable compared to other assessments. In the case of the Communicating and Responsiveness variables, the simple structure criterion was not met, with this variable showing quite high (>0.595) factor loadings for two isolated factors.

The majority of variables represented high communality estimates (>0.848); slightly lower values were observed for Sensitivity (0.760). The two-factor solution explained about 87% of the total variance.

## 7. Discussion

In the field of pedagogy, it is believed that proper referee–player interactions are the key to shaping the pro-social behavior and the multilateral development of young players. Kids achieve improved results in supportive environments. The task of building the right game climate also applies to the referee. The present research assumes that the educational function of a referee in youth sport is primarily to build positive relations with young players. The main aim of the research was to assess the quality of referee–players interactions during handball matches of children aged 9–12 years.

The exploratory factor analysis was made to examine the structure and interrelationships of the variables. Factor extraction starts with a model with one common factor [79]. The single-factor structure that contained all dimensions explained 71% of the variance. Results of the EFA suggests that the R-PIASS items are unidimensional. Only Positive climate variable loadings of Factor 1 at a lower level (0.573) but according to Hair et al. [80] loadings ±0.50 or greater can be considered as practically significant. Although in the social sciences, where information is very often less precise, it is not uncommon to consider a solution that accounts for 60% of the total variance as satisfactory [79,80], it was also decided to examine the two-factor structure. Then Factor 1 included five variables, apart from Positive climate. This can be easily explained. According to MacMahon et al. [43], managing players’ behavior consists of three components: (1) game organization (i.e., logistical arrangements of officiating event, preparation of a game); (2) decision-making appropriate for the game context; and (3) decision communication. In the tool used in our study, we also decided to include the Instructing component, because it fits the natural variability of the observed referee–players interactions. In this way we wanted to point out that the educational dimension of the referee’s work is also focused on providing information about game rules and teaching the value of sport. The study confirmed that Positive climate is a separate factor. This factor also includes Communicating (0.614) and Responsiveness (0.595). These variables were spread across two factors. This phenomenon (cross-loading) is probably due to the fact that both the content and the way of communicating the decision are crucial. The referee’s warm and calm voice contributes at the same time to building a positive climate on the pitch and a better understanding of the information he provides. The cross-loading of the Responsiveness variable can be explained by referring to Pianta et al. [71] who believe that an important element of emotional support and building a positive climate is the sensitivity to the child’s needs and the ability to accept his or her perspective. Other studies have shown that failure to take into account the needs of the child results in reduced learning outcomes [81]. It follows that the lack of ability to accept a child’s perspective reduces the chance of his or her development. The presence of the Responsiveness variable in Factor 1 can be explained by referring to a study by MacMahon et al. [43]. These authors emphasized that game management is a very capacious concept, which consists not only of managing the behavior of players, but also of analyzing the context of the event. The context determines both the steering style and how to react to players’ behavior. Reading and interpreting a player is essential for effective game management. Therefore, the Responsiveness dimension is also in Factor 1.

Considering the phenomenon of cross-loading in a two-factor structure, the fact that traditionally at least two or three variables must load on a factor to give a meaningful interpretation [82], and ease of interpretation, it was decided to adopt a one-factor structure.

### 7.1. Hypotheses Verification

The studies by MacMahon et al. [83], Simmons [53], and Dosseville, Laborde, Garncerzyk [37] indicate that a referee’s experience has a significant effect on his or her performance. The more experience a referee has, the better the quality and relevance of his or her decisions are. Usually, the players are very quick to know which referee they are dealing with. An inexperienced referee makes mistakes more often, gets nervous, panics, and imposes greater penalties while trying to stamp their authority. Young and inexperienced referees are at a disadvantage because in the opinion of the players they do not have qualities that the players value in referees. The players also declared that they would prefer that an inexperienced referee not officiate their matches [53]. The results of the research showed that, in terms of the educational function of the referee, greater experience did not translate into higher quality ratings of his or her interaction with players. There are two possible causes of this pattern: (1) The inexperienced (often young) referee is still full of enthusiasm, which is positively received by children, whereby such a referee can make up for the lack of experience with his or her enthusiasm; and (2) the lack of differences in ratings may be due to the fact that more experienced referees usually officiate the matches of older players and higher leagues. In youth sport, and certainly in adult sport, the educational function is less important, because the accuracy of decisions becomes the most important. A referee may be less open to the educational needs of a young athlete because of the habits he or she developed through officiating the matches played by older players. The lack of differences in the evaluation of the educational function of a referee between less and more experienced referees was also observed in studies of soccer referees [84]. The null hypothesis also assumed no differences in the referee’s assessment between close matches or matches with the advantage of one of the teams. This assumption was due to the fact that a close match could have caused more emotions of players and greater pressure on the referee. Therefore, the referee could have been more focused on controlling compliance with the rules of the game and less on the educational function. The results of the statistical analyses indicated that there were no grounds to reject the null hypothesis and therefore the result of the matches did not affect the assessment of the quality of their interactions with the players.

### 7.2. Positive Climate

The educational function of the referee requires that the match be a positive experience for the child. The results of the research show that the referees do not cope with this task. The quality of their interaction with the players in terms of building a positive climate has been assessed as poor. This was the lowest rated dimension. Referring this result to the description of this dimension (Table 2), an emotional distance between the referee and the players was observed during the matches and they were not interested in each other. The referees were sometimes in physical proximity to the players, but this was often not the case. Furthermore, they rarely smiled and were not enthusiastic about their duties and players. They did not talk to players about problems not related to the game (social conversation) and very rarely gave positive comments. Low ratings in this dimension do not prove that the referees do not work well. These results show that if referees are to support coaches and parents in achieving their educational goals, there is a space where they can improve. Similar conclusions from the research conducted among football referees were presented by Firek, Płoszaj, and Czechowski [84]. The results of the research by Fry and Gano-Overway [85] indicate that the athletes who experienced a caring climate in the sport environment had more joy from effort and were more committed. As a result, they gave up sport less frequently [86,87,88,89,90,91]. According to Anderson [35] and Fenoglio and Taylor [6], building a positive climate requires taking a different perspective on sport. In order to support the educational experience of the players, the referee should be less focused on competition and more on growth, enjoyment, and an inclusive engagement. This is particularly important due to the existing trend of children leaving sports, especially up to the age of 13 years [1]. The reason for this is excessive pressure from parents and coaches, lack of joy of exercising, pressure to win and compete, and negative experiences in sports relations [34,54,55].

### 7.3. Responsivness

The Responsiveness dimension was assessed as average, which in the context of the referee’s educational role means that the referees sometimes monitored the players for their needs for additional support although it happened that they did not do this. The referees noticed and responded to the needs of the players, but not to everyone. They did not always manage to solve problems and, consequently, it was extended over time. To respond to the needs of children, they must first be recognized. The ability to monitor players’ behavior and diagnose their needs is often considered a key skill of a referee. Cunningam [46] argues that sensitivity to the needs of players, understanding their motives and intentions, helps the referee to be more flexible, to better adapt to the context of the match, and to choose the refereeing strategy. At the same time, Cunningam adds that this skill is most difficult to teach. The referees themselves also admit that reading body language, applying active listening, and empathetic communication are necessary to guide the behavior of players [51]. The ability to anticipate players’ intentions and read their emotional states is one of the basic requirements for good refereeing [52,92,93].

### 7.4. Behavior Management and Proficiency

Two more dimensions: Behavior management ($\bar{x}$ = 4.7; SD = 1.5) and Proficiency ($\bar{x}$ = 5.0; SD = 1.5) were rated as average. The latter was the highest rated of all dimensions of the educational function of a referee. Of the referee surveyed, almost half received a good grade. This means that the referees are able to organize the game well and are well prepared to fulfil their duties. However, there are situations where there are unnecessary interruptions in the game and sometimes players do not know what to do. On the other hand, there were situations in Behavior management where the referees did not communicate their expectations to the players concerning players’ behavior during the game, or the rules they presented were not consistently enforced. The average quality level of the referee–players interaction in the Behavior management dimension means that referees sometimes monitored the players’ behavior in an attempt to prevent misbehaviors, but there have been periods without proactive actions on their part. It also happened that they tried to redirect negative behavior, but their strategies and techniques were not always effective, or the athletes solved disputes themselves. Episodes of inappropriate behavior could be observed during the matches, but they were short and limited to a small number of players. Similar results were obtained in studies on the quality of referee–players interaction during soccer matches [84]. The difference was that the soccer referees were rated higher in their ability to organize the game smoothly and without unnecessary interruptions. Simmons research [50] shows that only a small group of elite soccer referees use a wide range of techniques to ‘sell decisions’ and ‘minimize disruption to the game’. Raising the level of knowledge and skills of referees in this field is important for them to be able to overcome social tensions and negative behavior of athletes [94], rather than being the source of such problems [69,95]. Behavior management is also associated with the ability to manage game time. If the game is smooth, the referees express their readiness to communicate with the players, have the appropriate uniforms, then the players treat the referee with more respect and authority [48,53].

### 7.5. Instructing and Communicating

The study also evaluated the dimensions of Instructing ($\bar{x}$ = 4.5; SD = 1.6) and Communicating ($\bar{x}$ = 3.9; SD = 1.4) at a medium level. This means that if the situation required such measures, the referees clearly communicated the game rules and/or the values of sport. However, there were situations when they did not do this, or the athletes did not understand their instructions. Usually, the referees clearly signaled their decisions, but sometimes and/or some of them did so indistinctly or indecisively. From the standpoint of the athlete, a competent referee is one who gives explanations and instructions [46,48,49,52,96,97]. A study by Simmons [49] showed that when a referee communicates and appropriately explains his or her decisions (i.e., uses a calm but firm voice) the players perceive them as fairer. Such an attitude fosters building the referee’s authority, which is important from an educational point of view. A sports field can become a safe place for a child if the referee is a person the child trusts in every aspect of his or her work. Our research showed that referees communicate their decisions at a medium level. Therefore, this is another area where they can improve. Referees should communicate their decisions in a more noticeable and precise way because not all of them did this or they did this not always.

## 8. Conclusions

The studies of referees usually concern their place and role in professional sport. Few studies have examined their activities during competitions in youth sport. Inexperienced referees usually learn their profession and become proficient in sports for the youngest. It is therefore important to be aware that the child has specific emotional, social, cognitive, and health needs. The task of the referee is to diagnose and meet these needs. This approach is the essence of modern sport education aimed at providing a child with the positive experience that fosters their multifaceted development. A different role of a referee is evident in professional sport, where the main goal is to apply the laws of the game in a uniform and consistent way [98]. Therefore, it can be emphasized that what is good and expected in professional sport should not be automatically transferred by referees to youth sport. Some referees make the right decisions but do not support the educational process. There are also referees who may be wrong, but their attitudes and behavior on the pitch are a source of positive experiences for the players. It is also important that the referee should be aware of his or her shortcomings and areas where they can improve. This diagnosis allows them to take concrete actions to develop refereeing competences.

The sport environment is aware of the need for the education of professional referees who, together with coaches and parents, form a coherent educational system. The research provided empirical evidence that could be used as a basis for the modification of previous training programs for referees developed by local and national sports associations.

One of the purposes of the research was to develop a normative model of a referee–pedagogue who, through the appropriate quality of interactions with players, builds a positive, safe, and caring climate conducive to the multilateral development of the child and makes the sport a source of positive experiences. Thanks to this model, expressed in the R-PIASS tool, the referee can find out the behaviors required for the performance the educational function. The R-PIASS tool shows the educational role of the referee in many dimensions. 

The tool allowed for the evaluation of the referee’s work and its comparison to the assumed model. The results can be a source of information for individual referees and bodies responsible for training referees about the directions in which training programs should be modified. Undertaking such scientific research makes referees realize that their behaviour is significant for the emotional, social, and cognitive development of children. Taking into account the lowest-rated dimensions of referee–player interactions, the direct conclusions of the study are as follows:

- Referees’ training programs should include information about the emotional, social, and cognitive needs of children at various stages of their development;
- Referees should be made aware that they are an important link in the process of education through sport and that, together with the coach and parents of young athletes, they should build a caring climate on the handball court so that the sport can be a source of positive experiences for children, especially before the age of 13 (during this period, most children leave sports);
- Referees should be trained in building a positive climate, which consists in creating emotional ties with players (physical proximity, social conversation), expressed in an enthusiastic attitude and joy of contacts (smiling, engagement, positive affect reaction, positive comments, respectful and inclusive language, using players first names, listening to players). In addition, referees must be taught to actively monitor players’ emotional, cognitive, social, and health needs, as well as to respond to the players’ needs and solve problems.

The results of the research showed differences in the quality of referee–players educational interactions in soccer [71] and handball. Therefore, the referees of other team sports should also be analyzed to see if the quality of the referee’s educational activities depends on the type of team sport. The results of the examination did not show that the refereeing experience and the final result of the match affected the quality of referee–players educational interactions in soccer [71] and handball. This result should be confirmed based on a larger study group and in other team sports. Results also showed that referees focus more on controlling the rules of the game than on taking educational measures. It should be examined whether there is such a tendency in other team sports as well.

## Figures and Tables

**Table 1:** Dimensions of assessing the quality of referee–players interactions.

**Positive Climate**	**Responsiveness**	**Behavior Management**
Means the emotional bond between the referee and players, expressing mutual interest, enthusiastic attitudes and joy of contacts	Reflects the way the referee responds to the emotional, cognitive, social, and health needs of players	Concerns methods and techniques used by the referee to prevent and redirect negative behavior of players
**Proficiency**	**Instructing**	**Communicating**
Is the ability to organize the game smoothly and without interruptions and prepare the referee for refereeing	Refers to the way the referee provides knowledge, interprets the rules of the game, and teaches values in sport	Is a way of verbal and non-verbal communication of decisions by the referee

**Table 2:** Descriptions of dimensions.

**Positive Climate**			
**Indicators**	**Poor (1,2)**	**Average (3,4,5)**	**Good (6,7)**
Emotional connection (physical proximity, social conversation, the players seek support from the referee)	Clear physical and emotional distance between the referee and players is observed. In addition to the messages related to the game, the referee does not talk to the players.	It can be observed that the referee and the players show mutual interest, but this only applies to one team or individual players. A physical and emotional distance between the referee and the players is sometimes observed.	The referee shows great interest in all players. Physical contact and emotional closeness are observed. Their relationship is warm and supportive. The referee sometimes talks to the players about problems unrelated to the game.
Enthusiasm (smiling, engagement, positive affective reaction)	The referee does not show an enthusiastic attitude towards the players and his or her duties. They do not smile at all and do not reciprocate the positive emotions of the players.	The referee is enthusiastic and smiles, but there are moments when he or she does not do this or not to all players. The referee sometimes reciprocates the positive emotions of the players.	The referee shows enthusiastic attitudes and often smiles. He or she always reciprocates the positive emotions of the players.
Positive comments (verbal and non-verbal comments)	The referee does not give positive comments to the players at all.	The referee sometimes gives positive comments to the players or does it often, but they are apparently insincere. The referee gives positive comments to only one team or selected players.	The referee often gives positive comments to all players and they are apparently sincere and unforced.
Mutual respect (respectful and inclusive language, using players first names, calm voice listening to players)	The referee and players rarely, if ever, demonstrate respect for one another. Competitors do not recognize the authority of the referee, often questioning his or her decision.	The referee and players sometimes demonstrate respect for one another; however, these interactions are not consistently observed across time or players and it happens that the players question the referee’s authority.	The referee and players consistently demonstrate respect for one another. The referee has the authority and his/her decisions are not called into question.
**Responsiveness**			
**Indicators**	**Poor (1,2)**	**Average (3,4,5)**	**Good (6,7)**
Active monitoring of players’ emotional, cognitive, social, and health needs	The referee does not monitor the players to meet their needs and does not know when the players need additional support or help.	The referee sometimes monitors the players to meet their needs and notices when they need extra support or help, but there are moments when this does not happen.	The referee constantly monitors the players to meet their needs and always notices when they need additional support or help.
Responding to the players’ needs (fast meeting of the players’ needs)	The referee does not respond or neglects the players’ needs.	The referee sometimes responds to the players’ needs, or this reaction does not apply to everyone.	The referee always responds to the educational, social, emotional, and health needs of the players.
Solving problems	The referee cannot solve a problem that goes on and on.	The referee attempts to solve the problem, but he or she does not always do it effectively.	The referee manages to solve the problems that arise, and they do not last long.
**Behavior Management**			
**Indicators**	**Poor (1,2)**	**Average (3,4,5)**	**Good (6,7)**
Expressing expectations	The referee does not present (before the match) his or her expectations regarding the players’ behavior during the game.	The referee presents his or her expectations regarding the player behavior before the match, but not understandably or does not enforce them during the game.	The referee presents clearly and understandably his or her expectations for players’ behavior before the game and enforces them during the game.
Using preventative officiating	The referee does not attempt to prevent behavioral problems or does not notice the increasing negative climate.	The referee attempts to prevent behavioral problems on the pitch but does this not always or sometimes ineffectively.	The referee always attempts to prevent negative behavior and his or her actions are effective.
Redirecting negative behavior	The referee does not respond to the negative behavior of the players and it continues over time.	The referee responds to the negative behavior of the players, but his or her actions are not always effective, and the behavioral problems are extended over time.	The referee responds to the negative behavior of the players on an ongoing basis and his or her actions are effective, and the behavioral problems do not last.
**Proficiency**			
**Indicators**	**Poor (1,2)**	**Average (3,4,5)**	**Good (6,7)**
Continuity and flow of a game	The game is not smooth and there are unnecessary interruptions.	The game seems smooth, but there are sometimes unnecessary interruptions.	The game is smooth and there are no unnecessary interruptions.
Directing players	The game is not well organized, and the players often do not know what to do.	The game is well organized, but there are situations where players do not know what to do.	The game is well organized, and the players always know what to do.
Referee preparation (knowledge and skills, referee equipment)	The referee is not prepared to referee the match, does not have the appropriate uniform and equipment, or there are often situations where he or she seeks consultation or browses game rules.	The referee is prepared to referee the match, but there are occasions when he/her consults or looks into the game rules or does not have all the referee’s equipment.	The referee is well prepared to referee the match, has the appropriate uniform and full refereeing equipment.
**Instructing**			
**Indicators**	**Poor (1,2)**	**Average (3,4,5)**	**Good (6,7)**
Communicating the game rules and teaching the value of sport (clear and precise citation of game rules, effective explanation and instruction)	The referee does not instruct players and does not provide knowledge about the game rules and the values of sport.	The referee instructs players and provides knowledge about the game rules and the values of sport, but he or she either does so rarely or in a way that is not understandable for the players.	The referee instructs players and provides knowledge of the rules of the game and the values of the sport clearly and understandably.
**Communicating Decision**			
**Indicators**	**Poor (1,2)**	**Average (3,4,5)**	**Good (6,7)**
One-way communication techniques (decision communication e.g., whistle use and signaling, strong, sharp and visible signals)	The referee is either unclear or indecisive in communicating his or her decisions or does not communicate them at all.	The referee communicates his or her decisions, but not always in a clear, decisive, or visible way.	The referee communicates his or her decisions in a visible, clear, and decisive manner.

**Table 3:** The way to determine the assessment of each dimension.

Two Indicators	Three Indicators	Score	
P, P	P, P	1	POOR
P, A	P, P, A	2	
A, P	P, A, A	3	AVERAGE
A, A	A, A, A	4	
	P, A, G		
A, G	A, A, G	5	
G, A	A, G, G	6	GOOD
G, G	G, G, G	7	

Abbreviations: P = Poor; A = Average; G = Good.

**Table 4:** Differences in the assessment of the quality of referee–players interactions in all dimensions between experienced and inexperienced referees (*n* = 25).

Handball Referees						
Domains	Inexperienced (*n* = 17)		Experienced (*n* = 8)		Mann–Whitney U Test	
	Median	$\bar{x}$ Range	Median	$\bar{x}$ Range	Z	*p*-Value
Positive Climate	3.00	12.18	3.00	14.75	−0.888	0.375
Responsiveness	3.00	12.47	3.00	14.13	−0.545	0.596
Behavior Management	5.00	12.85	4.50	13.31	−0.148	0.882
Proficiency	6.00	13.29	5.00	12.38	−0.299	0.765
Instructing	4.00	13.38	4.00	12.19	−0.388	0.698
Communicating	4.00	12.74	3.50	13.56	−0.271	0.786

**Table 5:** Differences in the assessment of the quality of referee–players interactions in three domains between a close match and a match with the advantage of one of the teams (*n* = 25).

Results of the Games						
Domains	A Close Match (*n* = 17)		Advantage of One of the Teams (*n* = 8)		Mann–Whitney U Test	
	Median	$\bar{x}$ Range	Median	$\bar{x}$ Range	Z	*p*-Value
Positive Climate	3.00	9.00	3.00	9.00	0.00	1.00
Responsiveness	3.00	10.57	3.00	7.90	−1.11	0.265
Behavior Management	5.00	8.21	4.50	9.55	−0.549	0.583
Proficiency	6.00	9.14	5.00	8.90	−0.103	0.918
Instructing	4.00	7.79	4.00	9.85	−0.853	0.393
Communicating	4.00	9.29	3.50	8.80	−0.207	0.836

**Table 6:** Means (± standard deviation (SD)), median, and range values of the ratings of quality of referee–players interactions for individual dimensions observed in handball referees (*n* = 25).

Dimension	Mean (SD)	Median	Range
Positive Climate	2.8 (1.0)	3.0	1–6
Responsiveness	3.4 (1.2)	3.0	2–6
Behavior Management	4.7 (1.5)	5.0	1–7
Proficiency	5.0 (1.5)	5.0	2–7
Instructing	4.5 (1.6)	4.0	2–7
Communicating	3.9 (1.4)	4.0	2–7

**Table 7:** Results of factor analysis after Varimax rotation: values represent factor loadings for the assessments.

Variable	Factor 1
Positive Climate	0.573
Responsiveness	0.851
Behavior Management	0.930
Proficiency	0.878
Instructing	0.895
Communicating	0.903
Eigenvalue	4.305
Explained variance	71.753%

**Table 8:** Results of factor analysis after Varimax rotation (two-factor solution): values represent factor loadings for the assessments.

Variable	Factor 1	Factor 2
Positive Climate		0.950
Responsiveness	0.637	0.595
Behavior Management	0.903	
Proficiency	0.940	
Instructing	0.919	
Communicating	0.686	0.614
Eigenvalue	4.305	0.908
Explained variance	71.753%	15.134%
